# The Physical Characteristics of the Teeth

**Published:** 1896-12

**Authors:** Thos. Fletcher

**Affiliations:** Warrington, England.


ARTICLE VIII.

### THE PHYSICAL CHARACTERISTICS OF THE TEETH.

BY THOS. FLETCHER D. D. S., WARRINGTON, ENGLAND.

In the *Practitioner* for January is an article on some experiments by Dr. G. V. Black, detailing results which are neither curious nor unexpected, where they happen to be correctly stated. As an example of the want of ordinary care, may be taken the analysis on page 11, when "mercury" squeezed out of an amalgam is given to the second decimal, in ignorance of the elementary fact that "mercury" is not and cannot be squeezed out of any amalgam under any condition. It would be as easy to squeeze water out of sugar after they are mixed. The mass is simply divided into two amalgams, one containing enough mercury to keep it more or less fluid, and the other with a larger percentage of the solid metals. It will not yet be forgotten that I devoted the whole of my time for several years continuously to a series of exact experiments with filling material and alloys used for the purpose. My experiments were numbered by the thousands and I had plugs under continuous observation for periods extending over many years.

During the whole of this time I never succeeded in making a single plug, of any ordinary alloy, which could be considered satisfactory. If any excess of mercury had been added, the squeezing out of so-called mercury made the results so errotic that the system was entirely discarded at a very early stage. It is also stated on page 43 that "a block of amalgam made with sharp angles will preserve that form for an indefinite period, if it be put away in a box." This most certainly is not the case with the majority of amalgams in use at the present time, especially if they are made by the squeezing process. An approach to the shape is retained, but a flat surface made and polished as a flat mirror does not retain its flatness. Experiments made in ivory or in teeth are worthless, as regards permanent results. All my trials, for continued observations, were made in glass cavities of from three-eighths to one-half inch diameter.

So far as my experiments go, the system of mixing with excess of mercury and squeezing out, either with leather or during packing, is a great mistake, and the results are never to be depended on. An amalgam made from an alloy requires totally different treatment; the mercury used must be no more than is sufficient to make the surface of the grains cohesive; it must be built like cohesive gold. Any excess of mercury working up carries with it part of the alloy, rendering the plug irregular in texture and composition, and therefore unreliable. The removal of this excess may perhaps improve matters, but the irregularity of the result can never be prevented.

An amalgam, properly worked, like cohesive gold, and which will weld, but will not flow, is not liable to the changes which occur when other methods are used, and its value in the mouth can only be tested theoretically by packing in glass cavities of rather large diameter, at least equal in size to the largest plugs ever made in the mouth. That the squeezing-out process will make, in most cases, plugs which lasted several years in the mouth, is an undoubted fact, but this is not what a good operator would expect with a properly inserted amalgam of uniform texture and composition throughout, and it is undoubted facts that amalgams, treated in the manner they ought to be, will pack and make reliable sound plugs entirely under water; they will drive the water out and weld up sound and solid, retaining their shape permanently. That it is easier to daub a paste into a hole, rather than to build cohesive solid grains, is a fact which cannot be questioned, but this is, I think, not the point at issue. Hard grains, shaken in a glass tube with sufficient mercury to amalgamate their surfaces, can be packed and malleted like cohesive gold; and, unlike cohesive gold, this can be done wet or dry. The user of an amalgam should be a builder, not a plasterer.—*Dental Pract. and Advertiser.*

---

### Monthly Summary.

